# Application of immersive virtual reality mirror therapy for upper limb rehabilitation after stroke: a scoping review

**DOI:** 10.1007/s10072-024-07543-3

**Published:** 2024-04-29

**Authors:** Gdiom Gebreheat, Nick Antonopoulos, Alison Porter-Armstrong

**Affiliations:** 1 Healthcare Technologies Research Group, School of Health and Social Care (Sighthill Campus), Edinburgh Napier University, Edinburgh, UK; 2 Principal’s Office, Edinburgh Napier University, Edinburgh, UK

**Keywords:** Poststroke rehabilitation, Upper limb rehabilitation, Virtual reality, Mirror therapy, Immersive virtual reality mirror therapy

## Abstract

Mirror therapy is a commonly used rehabilitation intervention in post stroke upper limb rehabilitation. Despite many potential technological developments, mirror therapy is routinely delivered through the use of a static mirror or mirror box. This review aims to synthesise evidence on the application of immersive virtual reality mirror therapy (IVRMT) in poststroke upper limb rehabilitation. A scoping review was performed on relevant English studies published between 2013 to 2023. Literature search was undertaken on APA PsycInfo, CINAHL, Cochrane Library, MEDLINE, PubMed and Web of Science between August 5 and 17, 2023. Additional studies were included from Google Scholar and reference lists of identified articles. A total of 224 records were identified, of which 8 full-text articles were selected for review. All included studies were published between 2019 and 2023, and from high- and upper-middle-income nations. All the studies were experimental (*n* = 8). The total sample size in the studies was 259, most of whom were stroke patients with upper limb weakness (*n* = 184). This review identified three major themes and two sub-themes based on the contents of the studies conducted on the application of IVRMT: IVRMT’s technical application, feasibility and impact on clinical outcomes (motor recovery and adverse events). IVRMT was concluded to be a safe and feasible approach to post-stroke upper limb rehabilitation, offering enhanced engagement and motor recovery. However, more methodologically robust studies should be conducted to advance this area of practice, and to include a uniform IVRMT intervention protocol, dose, and use of outcome measure.

**Supplementary Information:**

The online version contains supplementary material available at 10.1007/s10072-024-07543-3.

## Introduction

In recent years, mirror box therapy has played an important part in the interventions offered by the healthcare provider in upper limb rehabilitation after stroke. Mirror therapy involves the use of a mirror that reflects the image of the unaffected upper limb in a way that the affected upper limb is visually replaced by its mirror image [1]. The mirror is positioned along the patient’s midsagittal plane and enables simple exercises and functional tasks with the unaffected upper limb to be performed while looking at the mirror. In this way, a visual illusion of the unaffected upper limb is created in place of the affected limb [2]. This visual illusion is believed to rewire neural connections in the brain [3] following repeated performance and exposures, and consequently can improve motor functioning of upper limbs in patients with stroke. Evidence from a Cochrane review also showed that mirror therapy had a significant positive effect on motor function and motor impairment and was found effective in improving pain and enhancing activities of daily living. However, it has been found that stroke patients’ adherence to mirror therapy across the treatment continuum has been observed to decline over time [4, 5]. It is postulated that users may tire of the simple movement protocols that have typically been used in mirror therapy intervention. Thus, the emergence of technologies such as immersive virtual reality may offer a more engaging environment for intervention delivery and is thus gaining attention as a promising technological innovation for post-stroke upper limb rehabilitation [6]. Furthermore, a recent study demonstrated the capability of immersive virtual reality-based interventions to stimulate neurons in a damaged brain areas and enhance functional recovery of upper limbs after stroke [7], eliciting multisensory stimulation [8] and its gamified nature has been effective in engaging stroke patients in upper limb rehabilitative process [9]. In a mechanism similar to that thought to be achieved through ‘traditional’ mirror therapy, Chang et al. recently confirmed that immersive virtual reality mirror therapy has the potential to enhance neuroplasticity in the motor cortex, that is, through neural reorganization and adaptation following injury which manifests in motor recovery in poststroke patients [10].

Therefore, the integration of mirror therapy principles with immersive virtual reality technology might present a synergetic and promising approach for upper limb rehabilitation delivery in poststroke survivors. This implies that using the concepts of mirror therapy in immersive VR technology could be a more effective and promising method for supporting stroke survivors regain movement and function in their arms and hands. The immersive and realistic nature of VR could potentially enhance the effects of mirror therapy, leading to better outcomes in rehabilitation. However, the landscape of existing evidence on this matter has not yet been collated. Therefore, the aim of this scoping review was to explore and synthesise the available evidence on the application of IVRMT for poststroke upper limb rehabilitation.

## Methods

A scoping review was performed to explore the nature, extent and characteristics of studies [11, 12] conducted on the application of IVRMT for upper limb rehabilitation following stroke (Supplementary file 1). To do this, the following five steps were adopted [12];

- **Step 1. Identifying the research question**
  The guiding review question was formulated according to the PICO (Population, Intervention, Comparison, Outcome) framework [13] and stated as “What is the available evidence on the application of immersive virtual reality mirror box therapy for upper limb rehabilitation following stroke?”.
- **Step 2. Identifying relevant studies**
  Once the authors established the review question, the review proceeded to identify relevant studies. The source of literature were online databases, related-article reference lists and Google scholar. A systematic literature search, limited to English-language articles published from 2013 to 2023, undertaken on APA PsycInfo, CINAHL, Cochrane Library, MEDLINE, PubMed and Web of Science between August 5 and 17, 2023. Furthermore, relevant studies were included from Google Scholar and related-article reference lists. The PICO framework was used as guidance in building literature searching strategies [13]. Accordingly, the following key concepts were used to formulate the literature search strategies; a) Immersive virtual reality OR virtual reality OR VR b) mirror therapy OR mirror visual feedback OR mirror box therapy OR mirror neurons c) upper limb OR upper extremity OR hand OR arm d) recovery OR rehabilitation e) stroke OR cerebrovascular accident OR CVA. Detail searching strategies and results can be seen in Supplementary file 2.
- **Step 3. Study selection**
  Two authors undertook the process of article screening and selection, using Mendeley software. All identified references were exported to Mendeley, duplicates removed, followed by title and abstract screening guided by the review aim and question. Full-text articles were evaluated for inclusion as per the following eligibility criteria:
  - Inclusion
    The PICO framework was used to refine the inclusion criteria [13].
    - *Population* -studies conducted on adult stroke populations with upper limb weakness.
    - *Intervention*-studies that applied immersive virtual reality mirror for upper limb rehabilitation post stroke.
    - *Comparison*- all studies with or without a comparator were eligible for inclusion.
    - *Outcome*- all studies needed to report on at least one outcome of clinical applicability relevant to use of the system.
  - Exclusion
    - Conference, abstract, registry, commentary and other opinion pieces were excluded.

Final inclusion of an article was decided upon through consensus of two authors (GG, APA) having thoroughly considered each study. Arbitration by the third author (NA) was not required as full consensus was achieved.

- **Step 4. Charting**
  Two authors (GG, APA) determined the specific information to be extracted from each included study and created a data extraction table to do so. Data that aligned with the review question was extracted and tabulated. This included details such as authors, publication year, country, methods, key findings, and other information pertinent to the broader review question. All authors reviewed and ensured the charting process was consistent.
- **Step 5. Collating, summarising, and reporting**
  This scoping review adapted a thematic analysis. Thematic analysis is a flexible approach that can be adapted to studies with a variety of questions and data types [14]. The analysis and synthesis of data was executed with NVIVO software. Initially, the selected articles were imported to NVIVO software, read to fully understood their content, and recurring concepts, ideas and meanings coded. These codes were then grouped as themes. Clear and meaningful names were given to the themes, which were organized as findings and reported coherently.

## Results

### Article selection process

**Fig. 1:**
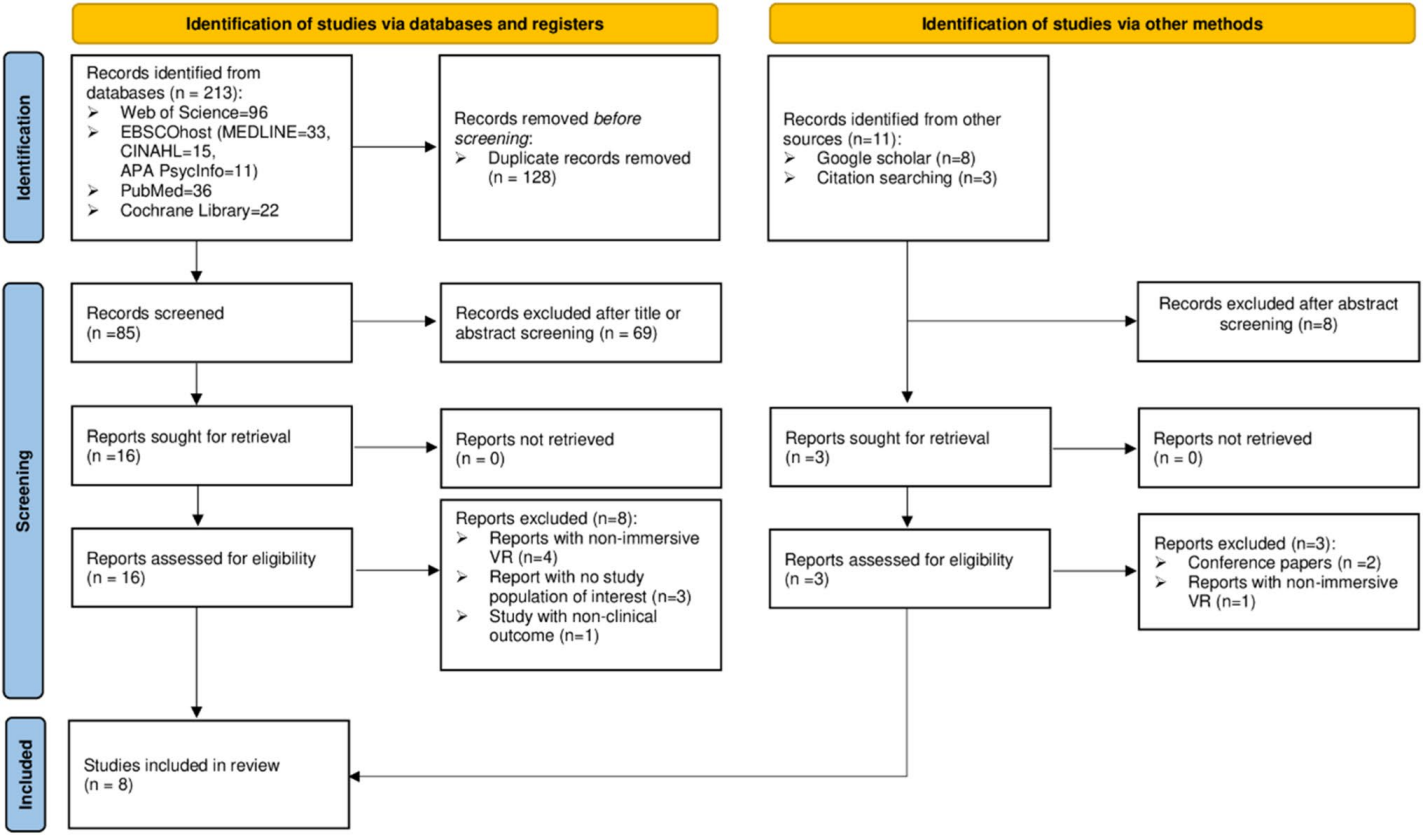
Flow diagram of selection of studies following the guidelines of the PRISMA statement 2020 [22]

As presented in Fig. 1, the search for articles generated a total of 224 records, of which 213 were retrieved through online database searching and the remaining 11 were handpicked records from Google scholar and related-article reference lists. From the database-generated records, 128 were removed for duplication, resulting in 85 records for further screening. Of these, 69 were excluded after title and abstract screening, and 16 full-text articles were retrieved and evaluated for inclusion in the review. In addition, out of 11 handpicked records, 3 full-text articles were retrieved and evaluated against the criteria for inclusion. Overall, 11 articles were excluded because of the following reasons: studies with non-immersive VR MT intervention (*n* = 5), reports with no study population of interest (*n* = 3), studies with non-clinically applicable outcome (*n* = 1) and conference paper (*n* = 1). Eight articles [7, 15–21] were identified as being eligible for inclusion.

### Characteristics of studies

The studies in this review were conducted in high- and upper-middle-income nations such as Germany (*n* = 2; 22,27), Taiwan (*n* = 2; 24,26), China (*n* = 1;14), Brazil (*n* = 1; 21), Poland (*n* = 1; 23) and USA (*n* = 1; 25). All the studies were published between 2019 and 2023, of which five were published in the past year 2022/23 [15–18, 21]. All studies were experimental (*n* = 8). Of these, three studies [7, 17, 20] had two comparison groups, two studies [15, 18] had three comparison groups, and the remaining three [16, 19, 21] had no comparison group. The total sample size in the studies was 259, ranging from 10 [19] to 54 participants [18], with the majority of subjects being stroke patients with upper limb weakness (*n* = 184). Most of the study participants were recruited from clinical settings, in particular, rehabilitation departments and centers (Table 1).

**Table 1:** The characteristics of included studies

Author, year, country	Aim	Method					Findings
		Study design	Setting	Participants & comparison groups	IVRMT application procedure	Outcomes and measurements	
(Jaques et al 2023), Brazil	To assess comparative perceived usability of immersive virtual reality mirror therapy (VR) and conventional mirror therapy (MT)	Experimental	Outpatient rehabilitation center	Stroke survivors aged between 30 and 80 (*n* = 15), stroke-free adults aged between 60 and 80 (*n* = 15), and young controls aged between 18 and 35 (*n* = 15)	Smartphone-compatible app was developed to provide a mirrored image of a body side. Participants used a smartphone with a working app attached to VR goggles, and then participants were instructed to carry out tasks with unaffected hands	Perceived usability (System Usability Scale (SUS)) and the NASA Task Load Index (NASA-TLX) and questionnaire)	In the stroke group, no significant difference of perceived usability was observed between MT and VR, as evidenced by the SUS and NASA-TLX. Both the MT and VR had low perceived usability. Furthermore, VR was less comfortable in comparison to the conventional mirror therapy
(Sip et al. 2023), Poland	To assess the application of immersive VR in post-stroke hand recovery	Experimental	Neurological rehabilitation department	20 first-episode stroke survivors aged between 40 & 64 (immersive VR mirror group (*n* = 10) versus conventional mirror group (*n* = 10))	An immersive VR application Virtual Mirror Hand 1.0 that uses sensor was intervened in stroke patients. Patients wearing Oculus Quest 2 experienced unreal virtual outer space, and hand movements were tracked using the sensor during exercise	36-Item Short Form Survey (SF-36), Fugl- Meyer Assessment Upper Extremity (FMA-UE)	From pre to posttest, both groups showed significant increment in the SF-36 and FMA-UE scores. Applying immersive Virtual Mirror Hand 1.0 treatment can enhance hand recovery after stroke, as noted by the mean score differences of FMA-UE and SF-36 from pre to posttest
(Chen, Kreidler, and Ochsenfahrt 2022), Germany	To assess the combined effect of mirror therapy and gamification concepts with VR for functional improvement in stroke patients	Experimental	Home-based	48 stroke patients aged between 31 and 90, (at least 2 months post stroke, 82.5% more than 12 months post stroke ‘chronic stage’)	A software called “Rehago” was used to implement the concept of mirror therapy in combination with gamified exercises into VR in stroke patients. A simulated mirror therapy mode was implemented in the Rehago with a standalone head-mounted display (HMD Pico Neo 2)	The Functional Independence Measurement score (FIM), Quality-of-Life score (EQ5D-5L)	The FIM significantly increased from baseline score of 101.48 ± 19.08 to 107.02 ± 16.32 at day 42. Similarly, the mean QoL score (EQ5D-5L) significantly increased from the baseline score of 69.65 ± 17.63 to 76.38 ± 13.61 at day 42
(Heinrich et al 2022), Germany	To assess the feasibility and psychophysical effects of immersive virtual reality-based mirror therapy in patients with upper limb paresis following first time stroke	Experimental	In-patient rehabilitation facility	11 people with upper limb paresis following first time stroke– mean time since stroke = 63.36 days (2 months approx.) 8 subacute 3 chronic (3 months +)	Participants carried out modified Berliner SpiegelTherapieprotokoll (BeST) mirror therapy protocol with an unaffected hand using an immersive VR system as adjunct to standard rehab. The protocol included mirror therapy performance standardization and documentation	**-**BeST documentation used to assess adherence, rehabilitation dose, tingling and progress - Simulator Sickness questionnaire and VR Adverse Event Monitoring questionnaire - FMA-UE - The meCUE 2.0 - The acceptance of technology and motivation questionnaire	The combination of an immersive VR system and mirror therapy protocol was feasible for clinical application. Psycho-physical effects, such as tingling or paresthesia, and hand functioning improvements were observed in 9 out of 11 participants, after the intervention. The average time used by the participants was 13.39 ± 3.03 min per session across the three interventions during the study. An average 13.93 ± 3.03 min rehabilitation dose was recorded across all interventions. Participants reported minimal negative effects such as tiredness after using the VR. Positive ratings of technological acceptance and motivation were recorded towards the immersive VR after the first and last interventions
(Hsu et al. 2022), Taiwan	To assess comparative effects of using VR-based mirror therapy (VR-MT), conventional occupational therapy (COT) and mirror therapy (MT)	Experimental (RCT)	Physical medicine and rehabilitation department	54 chronic stroke patients (6 months +) (allocated at 1:1:1 into COT, MT, VR-MR conditions)	50 min of occupational therapy care session was allocated to each condition. The session was compromised of 20 min of usual care and 30 min of the study condition. During the VR-MT treatment, stroke patients were instructed to look at the virtual movements of the affected hand through VR goggles that is- virtually mirrored image of the unaffected hand	FMA-UE, Semmes–Weinstein monofilament, Modified Ashworth scale (MAS), Motor activity log (MAL) and the box and block test (BBT) were recorded at pre-treatment, post-intervention, and 12-week follow-up	No significant group-by-time interaction effect was observed on the FMA-UE score but on the wrist sub-score of the FMA-UE and the BBT. The VR-MT had better effects on wrist training in comparison to the COT training. Plus, the VR-MT group had better UE coordination in comparison to the COT group and MT group at follow-up evaluation. With regards to the BBT score, the VR-MT group had better effect at post-intervention and follow-up evaluation, in comparison to MT group. In the VR-MT group, significant increments were observed from pre to posttest in both the BBT and the wrist component of the MAS scores
(Lin et al. 2021), Taiwan	To develop and assess performance of a virtual reality mirror therapy system for upper limb rehabilitation after stroke	Experimental	Stroke patients recruited from Department of Physical Medicine and Rehabilitation	-30 young healthy participants (MT (*n* = 15) versus (MTVR (*n* = 15)) -18 stroke patients (MT (*n* = 9) versus (MTVR (*n* = 9))	The healthy participants underwent two weeks of intervention sessions of VRMT (or traditional MT) Both groups in the stroke participants underwent 50 min/day, two days/week, for nine weeks of intense treatment. The VRMT group received 30 min of VRMT, followed by 20 min of regular motor task-specific training in every session of treatment. A participant wearing the VR goggle looks at a virtually mirrored hand which is tracked by the LMC from the movements of an unaffected side. Similarly, the MT group received 30 min of traditional MT, followed by 20 min of motor task-specific training in every session of treatment	-FMA-UE **In the healthy participants:** -The Pinch-Holding-Up Activity (PHUA) test was used to evaluate sensorimotor control of a hand -The Purdue Pegboard Test (PPT) for hand manual dexterity and bimanual coordination evaluation -The Minnesota Manual Dexterity test (MMDT) for gross motor coordination assessment	**Effects of VRMT on young healthy participants:** -The PHUA showed a significant treatment effect for the precision pinch performance in those who received VRMT -The PPT and MMDT showed no significant between-group differences in the results of hand function tests (for both hands), but significant within-group changes over time **Effects of VRMT on stroke patients with hemiplegia:** **-**A significant group-by-time interaction effect found on the total score of FMA-UE and hand part of FMA-UE **-**Better motor function improvements were seen among the VRMT group in comparison to the MT group -The FMA-UE hand component and total score showed significant improvement in the VRMT group -No significant within-group changes seen, over time, to the wrist component -No significant within-group differences detected in the total and sub-score of the FMA-UE test in the MT **-**No adverse effect reported
(Mekbib et al 2020), China	To design and implement VR protocol to provide limb mirroring exercises in an immersive virtual environment. It also aimed to assess treatment effect on brain reorganisation using resting state MRI	Experimental	Department of Rehabilitation Medicine (hospital)	23 (8 subacute stroke patients (average 38 days since onset) versus 15 healthy participants)	Patients received 8 h of VR training and 8 h conventional treatment over 2 weeks. The mirroring neuron virtual reality (MNVR)-Rehab system had different game-based exercises including reach-to-grasp tasks. The patient wearing the HMD immerses himself and navigates the virtual environment generated in the PC	FMA-UE	The stroke group underwent two weeks of VR-based limb mirroring therapy (VRLMT), showed moderate hand recovery. Furthermore, a significant VRLMT-induced change in functional connectivity (FC) values and FMA-UE score was recorded. The change of the bilateral M1-M1 FC value was positively correlated with the change of FMA-UE score. That means, recovery of UE increases as connectivity increases in relation to interhemispheric M1
(Weber et al. 2019), USA	To assess feasibility of immersive virtual reality (VR) mirror therapy for upper limb rehabilitation after stroke	Experimental	Community dwelling	10 chronic stroke patients with upper limb hemiparesis (6 months post stroke minimum) Average 81.9 months post stroke	The system compromised head mounted goggles (Occulus), and used the Wise Mind Software (Realiteer, CA), a novel adjustment for use and hand controller in unaffected hand and mirrored representation in system viewing as affected arm. A first-person view of virtual avatar adjusted to match personal characteristics such height, sex, and skin tone	**Feasibility assessed using:** -Patient compliance -Adverse event tracking -The SUS -The Simulator Sickness Questionnaire **Preliminary efficacy evaluated using:** -FMA- UE -Action Research Arm Test (ARAT)	The SUS mean score was 76 which indicates the system is usable. No adverse events were reported. No cyber sickness reported after sessions as evidenced by the simulator sickness evaluation. From pre to posttest, no significant changes were detected to the scores of FMA-UE and ARAT

This review identified three major themes and two sub-themes based on the contents of the studies conducted on the application of IVRMT: IVRMT’s technical application, feasibility and impact on clinical outcomes (motor recovery and adverse events).

### Technical application of IVRMT

The procedure of using IVRMT was similar across all the included studies. Seven studies developed an IVRMT system [7, 15–20] while one [21] relied on existing system. In all studies, IVRMT was implemented using smartphone or computer-compatible software or Apps that displayed a reversed image of the unaffected upper limb. The movement of the unaffected limb was captured by a Leap Motion Controller (LMC) and mirroring capability within the IVRMT software/App enabled duplication of these movements from the unaffected upper limb, leading to the creation of coordinated movements on the affected upper limb at the center of the VR goggles viewpoint. The IVRMT experienced by the participant is of both upper limbs represented within the VR goggles, and thus patients are immersed in the mirror illusion of bilateral activity and active use of the affected limb. The intervention delivered through the IVRMT primarily comprised training sessions incorporating exercises [16–20], functional activities [7, 15, 19, 21] and gamified virtual tasks [15, 21] (see Table 2). Gamified training sessions were dominant across the intervention protocols and were noted to be motivating and engaging for participants. However, the clinical intervention protocol and dose varied among all studies (Table 2) which thus inhibits any direct comparisons being made.

**Table 2:** Interventions and adverse events reported

Study	System	Intervention and dose	Outcome measures of adverse events	Reported adverse events
(Jaques et al. 2023), Brazil	Smartphone app, VR goggles,	Two tasks performed in a single session with the IVRMT 1) cube sorting task, fitting a geometric figure into an opening 2) Gridlock puzzle game, fitting as many pieces as possible in 5-min	Open ended questions to report any difficulties experienced	Nausea/dizziness, visual discomfort, headache, verbalized irritation
(Sip et al. 2023), Poland	The SciMed system, compromises the Virtual Mirror Hand 1.0 immersive VR application which is designed for use on the Oculus Quest 2 VR headset module	A protocol with five exercise component was intervened: 1) Flexion and extension of I-V fingers – opening and closing the fist, 2) Movement of spreading and bringing together I-V fingers, 3) Touching fingertips of II-V fingers with the thumb in sequence, 4) Dorsal and palmar flexion of hand in the wrist joint, 5) Supination and pronation of hand. Three series of exercises with 50 repetitions each was performed for every exercise type. This was carried out for 30 min and included 20-s breaks between the series of exercises over 18 days		Not investigated
(Chen, Kreidler, and Ochsenfahrt 2022), Germany	A standalone HMD Pico Neo 2, with preinstalled Rehago software with 10 gamified training	Rehago software developed for rehab based upon games eg: ball bouncing, labyrinth, asteroid blocking etc Simulated mirror mode with contralateral grip of controller (non-paretic side) 30-min training daily, 5 days/week over 6 weeks		Not investigated
(Heinrich et al 2022), Germany	A laptop computer-based IVRMT, with a Unity3D (v2017.3.0f3) software, Head-mounted display (Oculus CV1), LMC	Modified BeST mirror therapy protocol. Three sessions lasted 30-min each over 1 week. The protocol included hand movements using finger digits to convey numbers 1–5 with palm, wrist and arm in different positions. These included a) wrist extension/flexion, b) palm up/palm down, (c) elbow extension/flexion, and (d) hand sliding	Numeric and verbal pain Rating Scales, Adverse Event Monitoring questionnaire, Simulator Sickness Questionnaire	Minimal psycho-physical effect reported after VR use, such as tiredness, tingling sensation, or paraesthesia
(Hsu et al. 2022), Taiwan	Personal computer–based desktop IVRMT system with The Unity cross-platform game engine (used as a software), Leap Motion Controller (LMC) with 3 infrared light-emitting diode lights, 2 mono- chromatic infrared camera sensors, and an Oculus Rift (VR headset)	30-min IVRMT twice a week over nine weeks. This consisted of movements of forearm (such as supination/pronation, wrist extension/ flexion, finger extension/flexion) and hand (straight hand, hook fist, and full fist). 50 reps of hand movements	Adverse effects study report protocol	No adverse event (upper limb injury, dizziness, and visual disturbance attributable) reported
(Lin et al. 2021), Taiwan	Immersive VR system compromised of LMC, VR goggle (Oculus Rift), and IVRMT software	Seven hand- rehabilitation-related activities (a) supination/pronation, b) thumb-to-the-tip-of-the-finger movement, c) thumb circling, d) wrist flexion and extension, e) tendon gliding exercise (straight hand, hook fist, straight fist, and full fist), f) finger flexion & extension, g) key pinch. 50 reps/every movement over 30 min followed by 20 min regular motor-task specific training. Therefore, treatment was given on 50 min/day, two days/week, over nine weeks	Adverse effects study report protocol	No adverse event (dizziness, headache, nausea, or blurred vision) reported
(Mekbib et al 2020), China	The MNVR-Rehab system comprised of: (1) An HTC Vive head-mounted display (HMD) (2) two base stations to track the patient’s exact location in 3D; (3) LMC and (4) PC	IVR rehabilitation protocol consisted of carrying out reaching, grasping, and releasing tasks. Game based exercise selecting either unilateral or bilateral reach-to-grasp tasks of moving balls into a basket – 20 movements each session for 1 h/day, 4 days/week over 2 weeks		Not investigated
(Weber et al. 2019), USA	Head mounted goggles (Occulus), Laptop computer compatible-WiseMind Software®, Two tabletop infrared LED sensors, two hand controllers, fully immersive avatar system	A total of twelve virtual reality sessions over four weeks -Three 5-min treatment blocks performed 2X/session/30 min training -The three treatment blocks were: 1) exercise (5-min) 2) rock stacking (5-min), and 3) Functional tasks (5-min)	The Simulator Sickness Questionnaire	Little cybersickness, worsening symptoms

### Feasibility

Two studies exploring the feasibility of IVRMT for poststroke upper limb recovery [21], using the System Usability Scale (SUS) reported variations in usability. Jacques et al. [15] found that both mirror therapy and VR-based mirror therapy were both rated as having low levels of perceived useability. Whilst mirror therapy was viewed as being more easily adaptable and causing less overall discomfort, the comfort levels of the headset goggles was a key factor in useability of IVRMT for post stroke rehabilitation use and that IVRMT was viewed as requiring a greater effort with a less positive overall experience for older adults. Conversely, Weber et al. [19] concluded that IVRMT was well tolerated and safe, with high compliance and an above average SUS score.

One study [16] explored feasibility through use of the Acceptance of Technology and Motivation Questionnaire and the meCUE 2.0 user experience questionnaire. Results reported positive ratings towards IVRMT on both outcome measures with an overall adherence to the intervention of 100% and both participants and therapists reporting high ratings for usefulness and useability.

Two studies [17, 21] used standardized outcome assessments to assess impact upon quality of life through the use of IVRMT using the SF-36 [17] and the EQ-5D-5L scale [21]. Both studies reported statistically significant improvements in quality-of-life measurements following intervention.

### Impact on clinical outcomes

#### Motor recovery

Seven studies [7, 16–21] assessed the motor function recovery of stroke patients after IVMRT use. Six studies used the Fugl-Meyer motor assessment for upper extremity (FMA-UE) to evaluate upper limb motor function recovery of stroke patients after IVMRT interventions [7, 16–20]. One study [21] used the Functional Independence Measure (FIM) to measure the patients’ level of independence in performing various activities such as self-care. In six studies [7, 16–18, 20, 21] patients showed improvements to the recovery of their upper extremities following use of the IVRMT, of which, five studies [7, 16–18, 20] demonstrated statistically significant changes of FMA-UE scores pre- to post- tests, and the remaining study demonstrating a significant increase in baseline FIM score over a period of 6 weeks [21]. However, one study [19] demonstrated no such significant changes to the FMA-UE or to Action Research Arm Test (ARAT) scores.

One study suggested that IVRMT had a comparatively better effect on motor functioning of upper extremities over traditional mirror therapy [20]. Conversely, in another study, the comparative group-by-time difference of motor function between conventional occupational therapy, mirror therapy (MT), and VR-based MT was statistically insignificant (the FMA-UE total score) [18]. However, over the intervention period, significant within-group differences were recorded in both MT and VR-based MT groups, as evidenced by the total FMA-UE scores, but remained below the minimal detectable change [18].

Mekbib et al. [7] studied the mechanism of motor cortex and parietal cortex activity using mirrored VR activity. As a result, significant neural functional connectivity and functional improvements were noted. The researchers concluded that limb mirroring exercise in an immersive virtual environment was associated with cortical reorganisation, which significantly linked with motor functioning improvements.

#### Adverse events

Cybersickness is a commonly reported concern regarding the use of virtual reality in all settings [23]. Adverse events associated with the use of the immersive virtual reality by stroke patients with upper limb weakness was explored in five of the papers [16–20] (Table 2). The approach and tools adapted in the assessment of adverse outcomes varied across studies. Standardised outcome assessment was only undertaken in two studies using the Simulator Sickness Questionnaire [16, 19]; with adverse event monitoring questionnaires and protocols being used in four studies [15, 16, 18, 20] to assess more general susceptibility to symptoms such as finger tingling/numbness, heat sensation, fingertips sensation, dizziness, nausea, visual disturbance, unusual pain or feeling of tiredness.

Whilst all studies concluded that IVRMT was a well-tolerated and safe intervention for stroke patients with upper limb weakness, 3 studies did record some report of symptoms aligned with cybersickness [15, 16, 19]. Whilst these were small scale studies using mainly non-standardised outcomes, it remains an important aspect to explore further particularly given the likely overlay of post-stroke fatigue, sensory, perceptual and cognitive symptoms that this patient population present with. Interestingly, one study noted the report of amelioration in pain and sensory impressions after the use of IVRMT [17].

## Discussion

The aim of this scoping review was to explore and synthesise existing evidence on the application of immersive virtual reality mirror therapy for poststroke upper limb rehabilitation. Accordingly, eight studies were found that assessed the application of immersive virtual reality mirror therapy for upper limb rehabilitation in people with stroke. Amongst these, the earliest published research paper to use an avatar based immersional VR system was conducted by Weber et al. in 2019 [19] which was a small-scale study that concluded that the IVRMT was safe, and well-tolerated. All subsequent studies identified through this review adapted a variety of technology and software to develop an iteration of immersive virtual reality mirror therapy (Tables 1 and 2). Like the variation in technology, the dose, type and duration of the interventions were similarly varied across the studies as seen in Table 2. The duration of intervention ranged from two [7, 16] to nine [18, 20] weeks, and the basis of intervention founded on virtual games [15, 21], exercises [16–20] or functional tasks [7, 15, 19, 21]. Whilst this heterogeneity provides a broad lens through which to view the potential development and application of IVRMT, it highlights the need for the implementation of a more consistent technology, intervention and dose protocol to enable direct comparison across studies to be made.

In addition, immersive virtual reality mirror therapy was reported as a safe, tolerated, and feasible intervention for stroke patients with upper limb weakness [16–20]. However, adverse events such as cybersickness were not rigorously investigated and thus attention needs to be given to this aspect, particularly given the likely overlay of health issues related to a post stroke presentation that could be exacerbated by the use of an immersional VR-based system [23].

Nevertheless, six of the selected studies [7, 16–18, 20, 21] confirmed progressive upper limb recovery among stroke patients following immersive virtual reality mirror therapy. Of these, two studies [18, 20] presented the comparative effectiveness of IVRMT on motor recovery of upper extremities over conventional mirror therapy. However, while one of these studies concluded that IVRMT was statistically superior to conventional mirror therapy, the second did not find a significant difference between them. Furthermore, only one study has investigated and confirmed that IVRMT was less comfortable than conventional mirror therapy [15]. Of course, this discomfort might be due to the headset and motion sickness often associated with immersive virtual reality. Hence, further comparative studies are needed to conclude whether IVRMT is more effective for regaining motor recovery of upper extremities than conventional mirror therapy in upper limb rehabilitation after stroke. Moreover, it is evident that the effectiveness of rehabilitation programme relies upon the degree of patient engagement with prescribed interventions. In this application, engagement may have been enhanced through the multisensory experiences and illusive engagement of users provided by virtual reality-based mirror therapy [24].

### Limitations of the review

This review was restricted to articles published in the English language between 2013 and 2023 and accessed through the specified databases. Therefore, research papers published in other databases, outside this period and/or in other languages will have been omitted.

## Conclusion

This review found only eight studies published since 2019 that investigated the application of immersive virtual reality mirror therapy for poststroke upper limb rehabilitation. These studies focused on the application of immersive virtual reality-based mirror therapy and considered some key outcome measures such as feasibility, motor functioning and adverse events. Overall, immersive virtual reality mirror therapy was found to offer a safe and feasible approach to post-stroke upper limb rehabilitation, with enhanced participant engagement and improved motor recovery outcomes. However, the current scoping review has highlighted inconsistencies across studies in terms of intervention protocol type and dose, as well a lack of standardized measurement of cybersickness as a potential outcome. Furthermore, methodologically robust studies need to be conducted using a standardized protocol with a core outcome measure set to enable definitive conclusions to be drawn as to the patient benefits of immersive virtual mirror therapy for poststroke upper limb rehabilitation.

### Supplementary Information

Below is the link to the electronic supplementary material.

Supplementary file1 (DOCX 108 KB)

Supplementary file2 (DOCX 23 KB)

## Data Availability

All materials used to support the conclusions are submitted along with the manuscript.

## References

[1] Zhuang J-Y, Ding L, Shu B-B, Chen D, Jia J (2021) Associated mirror therapy enhances motor recovery of the upper extremity and daily function after stroke: A randomized control study. Hua X-Y, editor. Neural Plast [Internet]. Sep 29;2021:1–9. Available from: https://www.hindawi.com/journals/np/2021/7266263/10.1155/2021/7266263 PMC8494575 34630560

[2] Dohle C, Altschuler E, Ramachandran VS (2020) Chapter 20 - mirror therapy. In: Sathian K, Ramachandran VSBT-MP (eds) Academic Press, pp 449–461. 10.1016/B978-0-12-812492-5.00020-6

[3] Ramachandran VS (2005) Plasticity and functional recovery in neurology. Clin Med J R Coll Phys London 5(4):368–373 10.7861/clinmedicine.5-4-368 PMC4954210 16138492

[4] Rothgangel A, Braun S, Smeets R, Beurskens A (2019) Feasibility of a traditional and teletreatment approach to mirror therapy in patients with phantom limb pain: a process evaluation performed alongside a randomized controlled trial. Clin Rehabil 33(10):1649–1660 31066315 10.1177/0269215519846539

[5] Rothgangel A, Braun S, Winkens B, Beurskens A, Smeets R (2018) Traditional and augmented reality mirror therapy for patients with chronic phantom limb pain (PACT study): results of a three-group, multicentre single-blind randomized controlled trial. Clin Rehabil 32(12):1591–1608 30012007 10.1177/0269215518785948

[6] Demeco, A. Zola L, Frizziero A, Martini C, Palumbo A, Foresti R, Costantino C (2023) Immersive virtual reality in post-stroke rehabilitation: a systematic review. Sensors (Basel, Switzerland) 23(3). 10.3390/s23031712 10.3390/s23031712 PMC9919580 36772757

[7] Mekbib DB, Zhao Z, Wang J, Xu B, Zhang L, Cheng R et al (2020) Proactive motor functional recovery following immersive virtual reality-based limb mirroring therapy in patients with subacute stroke. Neurotherapeutics 17(4):1919–1930 32671578 10.1007/s13311-020-00882-x PMC7851292

[8] Giroux M, Barra J, Zrelli IE, Barraud PA, Cian C, Guerraz M (2018) The respective contributions of visual and proprioceptive afferents to the mirror illusion in virtual reality. PLoS ONE 13(8):1–14 10.1371/journal.pone.0203086 PMC6117048 30161207

[9] Gueye T, Dedkova M, Rogalewicz V, Grunerova-Lippertova M, Angerova Y (2021) Early post-stroke rehabilitation for upper limb motor function using virtual reality and exoskeleton: equally efficient in older patients. Neurol Neurochir Pol [Internet] 55(1):91–6. Available from: https://journals.viamedica.pl/neurologia_neurochirurgia_polska/article/view/70645 10.5603/PJNNS.a2020.0096 33314016

[10] Chang WK, Lim H, Park SH, Lim C, Paik N-J, Kim W-S et al (2023) Effect of immersive virtual mirror visual feedback on Mu suppression and coherence in motor and parietal cortex in stroke. Sci Rep [Internet] 13(1):12514. Available from: https://login.napier.idm.oclc.org/login?url=https://search.ebscohost.com/login.aspx?direct=true&db=cmedm&AN=37532803&site=ehost-live 10.1038/s41598-023-38749-8 PMC10397282 37532803

[11] Levac D, Colquhoun H, O’Brien KK (2010) Scoping studies: Advancing the methodology. Implement Sci 5(1):1–9 20854677 10.1186/1748-5908-5-69 PMC2954944

[12] Arksey H, O’Malley L (2005) Scoping studies: Towards a methodological framework. Int J Soc Res Methodol Theory Pract 8(1):19–32 10.1080/1364557032000119616

[13] McKenzie JE, Brennan SE, Ryan RE, Thomson HJ, Johnston RV, Thomas J (2019) Defining the criteria for including studies and how they will be grouped for the synthesis. Cochrane handbook for systematic reviews of interventions 33–65

[14] Braun V, Clarke V (2006) Using thematic analysis in psychology. Qual Res Psychol 3(2):77–101 10.1191/1478088706qp063oa

[15] Jaques ED, Figueiredo AI, Schiavo A, Loss BP, da Silveira GH, Sangalli VA et al (2023) Conventional mirror therapy versus immersive virtual reality mirror therapy: The perceived usability after stroke. Stroke Res Treat [Internet]. 2023 May 25;2023:1–8. Available from: https://login.napier.idm.oclc.org/login?url=https://search.ebscohost.com/login.aspx?direct=true&db=c8h&AN=163948627&site=ehost-live 10.1155/2023/5080699 PMC10234727 37275507

[16] Heinrich C, Morkisch N, Langlotz T, Regenbrecht H, Dohle C (2022) Feasibility and psychophysical effects of immersive virtual reality-based mirror therapy. J NeuroEngineering Rehabil [Internet] 19(1):1–20. Available from: https://login.napier.idm.oclc.org/login?url=https://search.ebscohost.com/login.aspx?direct=true&db=c8h&AN=159547976&site=ehost-live 10.1186/s12984-022-01086-4 PMC9540740 36207720

[17] Sip P, Kozłowska M, Czysz D, Daroszewski P, Lisiński P (2023) Perspectives of motor functional upper extremity recovery with the use of immersive virtual reality in stroke patients. Sensors (Basel) [Internet] 23(2). Available from: https://login.napier.idm.oclc.org/login?url=https://search.ebscohost.com/login.aspx?direct=true&db=cmedm&AN=36679511&site=ehost-live 10.3390/s23020712 PMC9867444 36679511

[18] Hsu H-Y, Kuo L-C, Lin Y-C, Su F-C, Yang T-H, Lin C-W (2022) Effects of a virtual reality-based mirror therapy program on improving sensorimotor function of hands in chronic stroke patients: a randomized controlled trial. Neurorehabil Neural Repair 36(6):335–345 35341360 10.1177/15459683221081430

[19] Weber LM, Nilsen DM, Gillen G, Yoon J, Stein J (2019) Immersive virtual reality mirror therapy for upper limb recovery after stroke: a pilot study. Am J Phys Med Rehabil 98(9):783–788 30964752 10.1097/PHM.0000000000001190 PMC6697203

[20] Lin C-W, Kuo L-C, Lin Y-C, Su F-C, Lin Y-A, Hsu H-Y (2021) Development and testing of a virtual reality mirror therapy system for the sensorimotor performance of upper extremity: a pilot randomized controlled trial. IEEE Access 9:14725–14734 10.1109/ACCESS.2021.3050656

[21] Chen C-H, Kreidler T, Ochsenfahrt A (2022) Rehago - a home-based training app using virtual reality to improve functional performance of stroke patients with mirror therapy and gamification concept: a pilot study. Stud Health Technol Inform 292:91–95 35575855 10.3233/SHTI220330

[22] Page MJ, McKenzie JE, Bossuyt PM, Boutron I, Hoffmann TC, Mulrow CD et al (2020) The PRISMA 2020 statement: An updated guideline for reporting systematic reviews. BMJ 2021:372 10.1136/bmj.n71 PMC8005924 33782057

[23] Yildirim C (2020) Don’t make me sick: investigating the incidence of cybersickness in commercial virtual reality headsets. Virtual Real [Internet] 24(2):231–9. Available from: 10.1007/s10055-019-00401-0

[24] Perez-Marcos D, Bieler-Aeschlimann M, Serino A (2018) Virtual reality as a vehicle to empower motor-cognitive neurorehabilitation. Front Psychol [Internet] 9:2120. Available from: https://www.frontiersin.org/article/10.3389/fpsyg.2018.02120/full 10.3389/fpsyg.2018.02120 PMC6224455 30450069

