# Supplementary material for: Application of immersive virtual reality mirror therapy for upper limb rehabilitation after stroke: a scoping review
Source: Neurol Sci. 2024 Apr 29;45(9):4173–84. doi: 10.1007/s10072-024-07543-3 (PMC11306508; doi:10.1007/s10072-024-07543-3)
Supplement: Supplementary file 2 — Supplementary file2 (DOCX 23 KB) [file 10072_2024_7543_MOESM2_ESM.docx]

| **Database** | **Searching query** | **Filter applied** | **result** |
| --- | --- | --- | --- |
| PubMed | **((((((immersive virtual reality) OR (virtual reality)) OR (VR) AND (fft[Filter])) AND ((((mirror therapy) OR (mirror box therapy)) OR (mirror visual feedback)) OR (mirror neurons) AND (fft[Filter]))) AND ((((upper limb*) OR (upper extremit*)) OR (hand*)) OR (arm) AND (fft[Filter]))) AND ((recovery) OR (rehabilitation) AND (fft[Filter]))) AND (((stroke) OR (CVA)) OR (cerebrovascular accident) AND (fft[Filter]))** | **English, from 2013 – 2023**  **Date /05/08/2023**  **15:58** | 36 |
| Cochrane Library | (Immersive virtual reality OR virtual reality OR VR) in Title Abstract Keyword AND (Mirror therapy OR mirror visual feedback OR mirror box therapy OR mirror neurons) in Title Abstract Keyword AND (Upper limb* OR upper extremit* OR hand* OR arm) in Title Abstract Keyword AND (Recovery OR rehabilitation) in Title Abstract Keyword AND (Stroke OR Cerebrovascular accident OR CVA) in Title Abstract Keyword - (Word variations have been searched) | **English, from 2013 – 2023**  **Date /05/08/2023**  **23:25** | 22 |
| Web of science |  |  |  |
| #1 | **((ALL=(immersive virtual reality)) OR ALL=(virtual reality)) OR ALL=(VR)** |  | 213,877 |
| #2 | **(((ALL=(mirror therapy)) OR ALL=(Mirror visual feedback)) OR ALL=(Mirror box therapy)) OR ALL=(Mirror neurons )** |  | 11812 |
| #3 | **(((ALL=(Upper limb*)) OR ALL=(Upper extremit*)) OR ALL=(hand*)) OR ALL=(arm)** |  | 2,405,585 |
| #4 | **(ALL=(recovery )) OR ALL=(rehabilitation)** |  | 1,691,183 |
| #5 | **((ALL=(stroke )) OR ALL=(Cerebrovascular accident )) OR ALL=(CVA)** |  | 691,173 |
| #6 | **#1 AND #2 AND #3 AND #4 AND #5** | **English, from 2013 – 2023**  **Date /06/08/2023**  **15:50** | 96 |
| MEDLINE (EBSCOhost) | **( (Immersive virtual reality OR virtual reality OR VR) ) AND ( (Mirror therapy OR mirror visual feedback OR mirror box therapy OR mirror neurons) ) AND ( (Upper limb* OR upper extremit* OR hand* OR arm) ) AND ( (Recovery OR rehabilitation) ) AND ( (Stroke OR Cerebrovascular accident OR CVA) )** | **English, from 2013 – 2023**  **Date /06/08/2023**  **16:43** | 33 |
| CINAHL (EBSCOhost) | **( (Immersive virtual reality OR virtual reality OR VR) ) AND ( (Mirror therapy OR mirror visual feedback OR mirror box therapy OR mirror neurons) ) AND ( (Upper limb* OR upper extremit* OR hand* OR arm) ) AND ( (Recovery OR rehabilitation) ) AND ( (Stroke OR Cerebrovascular accident OR CVA) )** | **English, from 2013 – 2023**  **Date /06/08/2023**  **16:43** | 15 |
| APA Psycinfo (EBSCOhost) | **( (Immersive virtual reality OR virtual reality OR VR) ) AND ( (Mirror therapy OR mirror visual feedback OR mirror box therapy OR mirror neurons) ) AND ( (Upper limb* OR upper extremit* OR hand* OR arm) ) AND ( (Recovery OR rehabilitation) ) AND ( (Stroke OR Cerebrovascular accident OR CVA) )** | **English, from 2013 – 2023**  **Date /06/08/2023**  **16:43** | 11 |
| **Handpicked articles** |  |  |  |
| Google scholar |  |  | 8 |
| From list of references |  |  | 3 |
| Total |  |  | 224 |
| Included in review |  |  | 8 |
